# The Relationship between Adolescent Dating Violence and Risky Health Behavioral Outcomes

**DOI:** 10.3390/healthcare12151464

**Published:** 2024-07-23

**Authors:** Neha Saini, Shamya N. Smith, Manasicha Wongpaiboon, Vanessa B. Crowther, Sarah Buxbaum, Rima Tawk

**Affiliations:** 1 Department of Neurology, College of Medicine, University of Florida, Gainesville, FL 32611, USA; nehasaini413@ufl.edu; 2 College of Pharmacy and Pharmaceutical Sciences, Institute of Public Health, Florida A&M University, Tallahassee, FL 32307, USA; smithshamya1@gmail.com (S.N.S.); sarah.buxbaum@famu.edu (S.B.); 3 Florida State University College of Medicine, Tallahassee, FL 32304, USA; mw15y@med.fsu.edu; 4 School of Allied Health Sciences, Division of Health Care Management, Florida A&M University, Tallahassee, FL 32307, USA; vanessa.crowther@famu.edu

**Keywords:** dating violence, risky health behavioral outcomes, mental well-being, school performance, teen dating violence prevention programs

## Abstract

Dating violence is a serious public health issue among adolescents due to the detrimental short- and long-term consequences. The purpose of this study is to examine the relationship between adolescent dating violence (ADV) and adverse health behavioral outcomes related to substance abuse, mental health, and select risky health behaviors such as feeling unsafe, school performance, and inadequate sleep within the state of Florida. This study used data from the 2015 Youth Risk Behavior Survey (YRBS). The high school students represented a weighted total of 542,818 (*n* = 4301). Logistic regression analyses, stratified by gender, examined the relationship between ADV and health risk behaviors after adjusting for race and grade. Proportions of ADV were as follows: 3.1% of students reported being abused both physically and sexually; 3.4% reported being abused only physically; 3.9% reported being abused only sexually; and 89.6% were uninvolved. ADV was associated with almost all the health risk behavior outcomes studied, with a few exceptions. Experiencing both kinds of abuse held the highest odds ratio among the four mutually exclusive categories of ADV. The findings from this study could be helpful in identifying youths who demonstrate warning signs of ADV abuse and thus could provide opportunities for targeted preventive interventions.

## 1. Introduction

Adolescent dating violence (ADV) is defined as any intentional, psychological/emotional, physical, or sexual abuse that occurs between people involved in a romantic relationship [1]. According to the Centers for Disease Control and Prevention’s (CDC) most recent Youth Risk Behavior Survey (YRBS) conducted in 2019, 8.2% of adolescents reported experiencing physical dating violence (PDV), and 8.2% of adolescents reported experiencing sexual dating violence (SDV) [2]. In Florida specifically, 8.1% of high school students reported having experienced PDV, and 8.1% reported having experienced SDV [3]. These rates of ADV present a public health concern at both the national and state level due to the immediate and longitudinal physical health and adverse psychological health implications that occur as a result of ADV [4]. ADV victimization was also found to worsen pre-existing mental health conditions by late adolescence in addition to poor academic achievement. The increased risk of intimate partner violence (IPV) is concomitant to adverse health outcomes surrounding ADV [4,5]. This study addresses the relationship between ADV and substance abuse, mental health, and other risky health behavioral outcomes such as feeling unsafe on school property, school performance, and inadequate sleep at the Florida state level.

Relationships play a significant role in this period of life, with over 50% of children reported to have been in a dating relationship by the age of 15 [6]. Suleiman and Deardorff’s study reported 85% of adolescents having an interest in romantic relationships prior to entering high school, with another 36% of adolescents reporting being in a romantic relationship by 13 years of age and 70% of adolescents who have reported having one by 17 years of age [7,8]. Past studies have shown a positive correlation between adolescent dating quality and several parameters of well-being including elevated levels of positive affect, life satisfaction, self-development, and self-acceptance [9]. Similarly, low levels of quality, such as dating violence, were linked to negative effects that have potentially serious health risks [9,10].

The adolescent stage of life is a crucial developmental stage that marks the transition into adulthood with dramatic neurological and behavioral changes that can have long-lasting effects which carry into adulthood [11]. During adolescence, development occurs in regions of the brain such as the limbic system that are responsible for pleasure seeking and reward processing and emotional responses [11]. Concurrently, there are also changes taking place in the pre-frontal cortex during this period that are responsible for executive functions such as organization, decision making, planning, and impulse control [11]. Social experiences, especially affective ones, are significant in shaping brain networks via changes in executive and perceptual functioning in both adaptive and maladaptive manners during the adolescent developmental period [12]. Additionally, this developmental period is where most mood and behavioral disorders first appear [12]. Adolescents who experience violence victimization during this critical period of development experience higher rates of eating disorders, depressive symptoms, risky sexual behavior, substance use, and smoking in late adolescence as compared to their non-violence-exposed counterparts [13]. Moreover, adolescents who experienced ADV undergo worse mental health outcomes in late adolescence even when accounting for pre-existing mental health parameters such as depressive symptoms, self-harm, and suicide attempts [5]. The current literature surrounding ADV and substance abuse finds that adolescents who were victimized relied on alcohol and drugs to cope during and after the relationship [14]. A longitudinal study found that ADV victimization may lead to worsening of substance use including cigarette smoking, alcohol consumption, marijuana, and cocaine use in the long term [15,16]. Within Florida, alcohol and methamphetamine use among adolescents were associated with higher rates of ADV [17].

While much of the literature supports that the adverse effects of dating violence on adolescent health are multifaceted, what makes them even more concerning are the long-term implications and unintended consequences of these effects that carry into adulthood. Several studies have indicated the continuity of dating violence victimization in adolescence into intimate partner violence in adulthood, where these long-term health outcomes become evident [10]. The National Intimate Partner and Sexual Violence Survey, which is an ongoing nationally representative random sample digital dial telephone survey, found that 1 in 5 women and nearly 1 in 17 men who ever experienced rape, physical violence, and/or stalking by an intimate partner first experienced some form of intimate partner violence between 11 and 17 years of age [18]. Despite both men and women having experienced some form of ADV and IPV, males are far less likely to report and seek formal support services as compared to their female counterparts [19]. Women are more likely to experience physical abuse in addition to more severe injuries and distress, whereas men are more likely to experience psychological abuse [20].

The longitudinal health risks associated with experiencing physical partner violence by an intimate partner include frequent headaches, chronic pain, sleep disturbances, and poorer physical and mental health than those who have not experienced these forms of violence [18]. Women who had experienced PDV and SDV by an intimate partner were also more likely to report a wide array of adverse outcomes including adverse pregnancy outcomes, musculoskeletal problems, genitourinary problems, adverse mental health outcomes, somatic symptoms, cardiovascular problems, and disorders affecting the nervous system and brain [21]. Additionally, those who experienced these forms of violence had a higher prevalence of asthma, diabetes, and irritable bowel syndrome [18]. The longitudinal health risks associated with psychological partner violence include post-traumatic stress disorder, depression, and suicidal ideation. Psychological or emotional abuse was more impactful in leading to suicidal ideation and suicide attempts as compared to physical abuse, which highlights the significance of negative mental health outcomes as a result of IPV. Death by suicide accounts for 30% of IPV-related deaths; mediating mental health early in ADV can prevent future victimization of both physical and psychological abuse [22].

IPV poses an economic burden with an estimated lifetime cost of USD 3.6 trillion for 43 million adults with a history of victimization in the United States [23]. In total, USD 2.1 trillion is attributed to medical costs, USD 1.3 trillion is attributed to lost productivity to both victims and perpetrators, USD 73 billion to criminal justice costs, and USD 62 billion to victim property loss and damage [23]. Role functioning and health conditions exhibit a direct relationship where deteriorations in health can lead to limited role functioning, as seen in victims of ADV, further contributing to national economic burden.

Although the current literature has evaluated the association between dating violence amongst adolescents and various adverse health outcomes, almost all of these studies have been assessing national trends. Nationally, 23.2% of women and 13.9% of men have been victims of dating violence during their lifetime, whereas in Florida, 37.9% of women and 29.3% of men have endured some form of dating violence [24,25]. With Florida’s higher than national average rate of dating violence occurrence and the lack of focus on trends at the state level, this study fills in knowledge gaps about ADV trends that can mediate future IPV trends. The purpose of this study is to examine the relationship between ADV and adverse health outcomes related to substance abuse, mental health, and other select risky health behavioral outcomes such as feeling unsafe on school property, school performance, and inadequate sleep in Florida.

## 2. Methods

### 2.1. Participants

This study is based on data collected from the 2015 national Youth Risk Behavior Survey (YRBS) of high school students who are in grades 9–12. The YRBS is a national school-based cross-sectional survey that was first developed in 1990 to monitor priority health risk behaviors that contribute significantly to the leading causes of disability and death, as well as social problems among youth and young adults in the United States. The behaviors include those that contribute to unintentional injuries and violence, sexual behaviors that contribute to unintended pregnancies and STIs, and alcohol and drug use, as well as unhealthy dietary behaviors and inadequate physical activity. Florida Departments of Health and Education supervises the YRBS data in partnership with the Centers for Disease Control and Prevention [26]. Student participation in the survey is anonymous and voluntary. The students who participate in the survey report their answers on a self-administered questionnaire, in adherence with local parental consent procedures. The process consists of a three-stage cluster probability sample design. The survey is weighted to be representative of Florida public high school students. Sampling methods are applied to the data to adjust for the non-response and the oversampling of Black and Hispanic students. More details about the YRBS questionnaire are described elsewhere [27,28]. We accessed the Florida YRBS dataset by signing a data use agreement with the Florida Department of Health.

### 2.2. Variables

- Outcome variables. Outcome variables included the following: (1) substance use, (2) mental health risks, and (3) other select risky health behaviors. Substance use outcomes included tobacco, alcohol, or marijuana use. Mental health risks included suicide attempt and depression. The select risky health behaviors were school performance, feeling unsafe on school grounds, and inadequate sleep.

Tobacco, alcohol, and marijuana use were assessed with the questions: “During the past 30 days, on how many days did you smoke cigarettes?”, “During the past 30 days, on how many days did you have at least one drink of alcohol?”, and “During the past 30 days, how many days did you use marijuana?”, respectively. The mental health variables consisted of suicide attempt and depression. Suicide attempt was measured using the question: “During the past 12 months, how many times did you actually attempt suicide?” Depression was estimated with the question: “During the past 12 months, did you ever feel so sad or hopeless almost every day for two weeks or more in a row that you stopped doing some usual activities?” School performance was evaluated with the question: “During the past 12 months, how would you describe your grades in school?” The perception of safety was measured with the question: “During the past 30 days, on how many days did you not go to school because you felt you would be unsafe at school or on your way to or from school?” Sleeping was evaluated using the question: “On an average school night, how many hours of sleep do you get?” The responses for all these outcome variables were dichotomized for analyses.

- Independent variables. PDV and SDV were treated as independent variables. PDV was assessed with the question: “During the past 12 months, how many times did someone you were dating or going out with physically hurt you on purpose? (Count such things as being hit, slammed into something, or injured with an object or weapon)”. SDV was assessed with the question: “During the past 12 months, how many times did someone you were dating or going out with force you to do sexual things you did not want to do? (Count such things as kissing, touching, or being physically forced to have sexual intercourse)”. Consistent with prior approaches, a combined categorical variable was generated using the sexual and physical ADV questions resulting in four mutually exclusive dating violence categories based on the responses from the survey: physical abuse only, sexual abuse only, both kinds of abuse, or none [29,30]. Students who had missing data for either PDV or SDV questions were excluded from the analysis. Sociodemographic variables included age, race/ethnicity, and sex. Age was classified into 3 categories: 14 or younger, 15 to 17, and 18 or older. Race/ethnicity was categorized into 3 groups: White non-Hispanic, Black non-Hispanic, Hispanic.

### 2.3. Data Analysis

This analysis was stratified by sex, as previous research has shown that there are gender differences in the way males and females experience different types of dating violence [18,31]. Multiple logistic regression analysis was conducted after adjusting for race and grade to examine the relationship between ADV categories and the variety of adverse health outcomes (substance use, mental health, and select risky health behaviors factors). All analyses were conducted using PROC SURVEYFREQ and PROC SURVEYLOGISTIC in the statistical software SAS 9.4 (SAS Institute Inc., Cary, NC, USA). Data were weighed to adjust for varying probabilities of selection and non-response.

## 3. Results

### 3.1. Survey Respondents Characteristics

Table 1 describes the characteristics of the overall adolescent population. The high school student population represented a weighted total of 542,818 (*n* = 4301). Most students (74.9%) were between the ages of 15 and 17 in the weighed sample, 11.3% were 14 years old or younger, and 13.8% were 18 years old or older. Whites (49.2%) were the largest racial group, followed by 31% Hispanics, and 19.8% for Blacks. The female students who participated in the survey were 51.5%. With respect to grade level, 27.1% of students were in 9th grade, 26.4% in 10th grade, 24.1% in 11th grade, and 22.4% in 12th grade. Overall, 3.1% of students were involved in both kinds of abuse (PDV and SDV); 3.4% in physical abuse; 3.9% in sexual abuse; and 89.6% of students were uninvolved in abuse.

### 3.2. Association between Adolescent Dating Violence and Health Risk Behaviors

#### 3.2.1. Substance Use and Mental Health Factors

ADV was associated with almost all five substance use and mental health risk behaviors except for some differences (Table 2). The magnitude of effects across gender varied widely, to a large extent in some cases. Boys who experienced both kinds of abuse reported higher odds on three substance abuse-related behaviors than girls. For mental health outcomes, the odds of suicide attempt were strikingly higher (more than double in magnitude) among males who experienced both kinds of abuse as compared to females. Furthermore, suicide attempt held the highest odds ratio among all substance abuse and mental health-related behaviors among boys compared to girls (AOR = 34.221 and AOR = 15.199, respectively) with both kinds of abuse. Female students who were physically and sexually abused were about 16× more likely to attempt suicide as compared to those female students who did not experience any physical or sexual abuse after adjusting for race and grade. The second largest association in magnitude among substance use outcomes was for tobacco use for both male and female students experiencing both kinds of abuse (AOR = 9.338 and AOR = 8.953, respectively). Experiencing sexual abuse failed to reach statistical significance with tobacco use among males but not females. In contrast, depression had a stronger association among females experiencing both kinds of abuse than males (AOR = 4.387 and AOR = 3.834, respectively). Having been sexually abused only was associated with depression among females (AOR = 3.551) but not male students.

#### 3.2.2. Select Risky Health Behavioral Outcomes

Table 3 shows the association between abuse victimization and select risky health behavioral outcomes. Significant differences in magnitude between gender occurred for the outcome “not going to schools due to safety concerns”, where males who experienced both kinds of abuse had strikingly higher odds than females. Feeling unsafe had the strongest association among the select risky health behavioral outcomes for both kinds of abuse among male students compared to girls (AOR = 18.521 and AOR = 5.370, respectively). Female students were about 5.3× more likely to feel unsafe as compared to those female students who were uninvolved with any physical or sexual abuse.

Experiencing sexual abuse only was associated with poor school performance among female students only (AOR = 2.740). Females who experienced sexual abuse only were about 2.7× more likely to report a poor school performance compared to females who were uninvolved with any sexual or physical abuse. However, poor school performance was not associated with having been a victim of physical abuse only or both kinds of abuse among male and female students. Experiencing both kinds of abuse failed to reach statistical significance with school performance for both males and females. The odds ratios that were significant are bolded in Table 2 and Table 3.

Having been only physically abused was associated with inadequate sleep among males (AOR = 2.402) but not female students. Experiencing sexual abuse only was associated with inadequate sleep among females (AOR = 1.882) but not male students. Experiencing both kinds of abuse failed to reach statistical significance with inadequate sleep for both male and female students.

## 4. Discussion

This study aimed to explore the association between dating violence—within the parameters of physical abuse, sexual abuse, or both—and adverse health outcomes among teens in Florida after adjusting for race and grade level in school. Among the adverse outcomes including tobacco, alcohol, marijuana use, suicide attempts, and depression, suicide attempts had the highest odds ratio. Males were drastically more likely to commit a suicide attempt if they were victims of physical and sexual abuse, as compared to their female counterparts. Concerning the select risky health behavioral outcomes, males had the highest association between dating violence and feeling unsafe. The findings from this current research were consistent with previous studies. Black et al. in 2006 evaluated the association between physical dating violence and self-reported risk behaviors such as sexual activity, attempted suicide, current cigarette use, episodic heavy drinking, and physical fighting. All of these five risk behaviors, with the exception of current cigarette use, were significantly associated with PDV victimization [32]. This was consistent with our results in that ADV, specifically sexual abuse, had a statistically significant association with females and tobacco use. However, this relationship failed to reach statistical significance in males who experienced sexual abuse only within our study, which points to the need for further analysis in more recent YRBS datasets. Eaton et al. in 2007 found that adolescents who experienced dating violence victimization reported twice as much alcohol, illegal drug use, and cigarette use; however, we note that Black et al. reported that smoking was not significant. We found that our results were consistent with these previous studies but that males experienced higher odds on three substance abuse-related behaviors more than females. Moreover, PDV was specifically found to be associated with negative mental health outcomes, such as disordered eating and suicidal ideation [33]. Youth who reported being victims of relationship violence were more than twice (62%) as likely to consider, as well as attempt, suicide than those who did not report being assaulted by a boyfriend or girlfriend [34]. For our mental health outcomes data, the odds of suicide attempt were strikingly higher among males as compared to their female counterparts. Furthermore, the presence of suicide attempts held the highest odds ratio among all substance use- and mental health-related behaviors among males as compared to female, whereas depression had a stronger association among females as compared to males. Our results surrounding the presence of depression were consistent with another study that found an association among IPV and depressive symptoms, though this previous study did not examine sex differences [35]. Dating violence not only increases the risk for developing mental health concerns and high-risk behaviors but also affects adolescents academically. Current studies show a notable increase in the prevalence of dating violence being associated with poorer grades in school, with the lowest prevalence among adolescents earning mostly As and the highest among those earning mostly Cs, Ds, and Fs [33]. This was not consistent with our study, as we found no association between ADV and poor academic performance except for female students who experienced sexual abuse only.

A survey carried out by Eaton et al. [33] reported a positive association between alcohol and marijuana use and dating violence. Previous research found that dating violence perpetration and victimization are linked to suicide attempts [36]. Similarly, while research by Fletcher on the effects of intimate partner violence on health in young adulthood in the US did not distinguish the impact of intimate partner violence by gender, the study found an association between IPV and depressive symptoms [37]. Furthermore, our study results are consistent with the findings of a 2013 longitudinal study of adolescent health by Exner-Cortens, Eckenrode, and Rothman, who found increased heavy drinking, depression, and suicidal ideation among female victims and increased suicidal ideation and marijuana use among male victims compared to participants who did not report teen dating violence victimization [38]. Historically, males were primarily portrayed as perpetrators; however, recent epidemiological data have demonstrated that high-risk male adolescents are at equal risk as females of becoming victimized [39]. The data displayed that more male adolescents reported having experienced SDV as compared to their female counterparts [22] and unveiled not only the ubiquitous nature of ADV but also how it can disproportionately affect the male adolescent population [39,40]. The differences in magnitude of outcomes as previously described between sexes are consistent with the findings of our study. Certain variables had statistically significant associations for one sex but not the other while other variables had differences in magnitude between sexes. Further implications for research can involve assessing what contributes to these sex differences and if they are present across a lifetime.

Furthermore, adverse childhood experiences (ACEs) should be accounted for as risk factors, as the recent literature has linked a significant association with IPV victimization in adulthood, in addition to its potential as a confounding variable [41]. Although ACE is not the sole environmental component that predisposes adolescents to ADV and IPV, adolescents are still at greater risk of dating violence victimization and perpetration at all ages. ACEs including physical, emotional, and sexual abuse, physical and emotional neglect, interparental violence, parental substance abuse, and low parental warmth were all significantly associated with dating violence victimization [41]. Recommendations for future studies should include the different types of ACEs as independent variables to examine how each contributes as a risk factor for both ADV and IPV.

### Limitations

Our findings may not be representative at the national level for either other states’ public schools or other types of schools in Florida such as magnet or private schools, while also being unable to take into account high school youth who had dropped out. In addition, the narrow age range limits the study’s ability to be applied to other age groups. The YRBS data may not factor in under- or over-reporting. Due to the nature of YRBS being a cross-sectional study, temporal relationships could not be established, and the presence of pre-existing psychological variables could not be controlled for.

## 5. Implications for Public Health Practice

In 2010, the Florida Statute 1006.148 was created and required each district school board to adopt policies that prevent teen dating violence and abuse while also providing training for schoolteachers, staff, and administrators on how to apply these policies into the curriculum. Nonetheless, national rates of ADV have steadily increased between 2017 and 2019, with national prevalence estimates of 8.2% of high school students having experienced sexual dating violence and 8.2% having experienced physical dating violence within the last year [2,41]. Although Florida is one of 30 states with dating violence school policies, the types of prevention programs available throughout the state and on a national level have varied within the current literature [42]. Efforts to not only reduce ADV but prevent revictimization should not be limited to school but also integrated into the community. Healthy People 2030’s objective of reducing sexual or physical adolescent dating violence can be achieved by mandating the expansion and standardization of school sexual and health education throughout school districts by the following methods [43]:

- Promoting high school prevention programs that are integrated into classroom curriculum and thus prevent the initiation or reduce ADV perpetration and/or victimization in areas of psychological, physical, and sexual abuse.
- Administering a social–emotional school-based prevention program that incorporates a universal-level intervention approach where students are educated on conflict-management skills, knowledge of ADV, healthy relationship skills, and empowerment of bystander reporting.
- Incorporating ADV training for school psychologists/counselors as part of their continuing education in order to identify ADV and respond appropriately. This should be further broken down into more emphasis on certain screenings and/or interventions based on sex. For example, males historically seek less formal support following dating violence but were shown to have double the magnitude in suicide attempts in our study as compared to females. School clinicians should follow adolescent males who have been victims of dating violence closely in this mental health context due to their nature to utilize less formal services and help.
- Referring youth who are at-risk of becoming victims of IPV or abusive relationships to community-based prevention programs and referring victims to community-based victimization prevention programs to reduce revictimization. Youth who are not intervened with and do not receive help from support systems can become at risk for revictimization and can become at risk for mental health outcomes such as suicide attempts and depression in addition to substance use, as discussed in our study.
- Establishing parent/caregiver-based programs that educate the learner about ADV, substance use, and mental health. This should be integrated alongside school programs that focus on the academic components of school performance that can become negatively affected as seen in our study. For example, adolescents within our study reported “not going to schools due to safety concern” and “feeling unsafe”. Creating programs that target how to address these concerns based on the type of dating violence (physical or psychological) can allow for individualized solutions that improve students’ motivation and perception of safety in attending school.

Most programs have been aimed at ADV perpetration and victimization with evaluations based on outcomes related to sexual and physical abuse frequency, but little evidence is available on how these programs affect the psychological component of adolescents [44]. Moreover, many current programs are generalized with little emphasis on individualized adolescent plans. The results from this current study emphasize the need for programs that additionally target the psychological component of both perpetrators and victimization. In the context of social factors, peer relationships are interdependent and promote a significant but unique impact [45].

Prosocial behaviors and healthy peer relationships can serve as a protective factor in not only preventing ADV but in influencing adolescents to find romantic partners who also fit this narrative [46]. As a result, programs that incorporate students’ social environments at both the school-based level and community level should be encouraged. Although current prevention programs have been successful in gaining positive results, most of them target immediate changes with scarce literature on how these programs affect the sustainability of these positive behaviors [47]. Current parent/caregiver-based ADV programs are scarce, as those that currently exist target specific risky behaviors individually such as substance use, suicidal behavior, or sexual risk behavior without integrating ADV and how it relates to these risk behaviors [48]. A parent/caregiver-based program known as Teach One Reach One focuses on improving relationships and sexual health among adolescents and has been found to reveal significantly lower rates of dating violence acceptance while increasing rates of self-efficacy in dating violence avoidance [48]. Sessions are comprised of a program instructor segment and parent and child integration followed by an acquired skills practice session. A program structure similar to this with more focus on ADV and negative acute health implications such as those found in this study (mental health, substance use, risky health behaviors) can improve adolescent dating health.

To generate sustainable and longitudinal preventative behavioral changes, ADV intervention programs should address multifactorial components that include individual, relationship, community, and society factors [49]. Policymakers should secure funding on programs that are targeted at preventing ADV and victimization at multiple levels, with an emphasis on expanding content about shared risk and protective factors. Early prevention is associated with lower costs as compared to reactive interventions such as healthcare costs, legal costs, lost earnings, or indirect losses due to affected psychological functioning [49,50].

## 6. Conclusions

ADV poses a public health concern due to its immediate and lasting adverse health outcomes. Adolescents who undergo violence victimization during this vital phase of development experience depressive symptoms, suicide attempts, tobacco use, alcohol consumption, drug abuse, and perceptions of unsafety. ADV intervention programs should tackle multifactorial components that incorporate individual, peer healthy relationships, community, and society factors.

## Figures and Tables

**Table 1:** Characteristics of Respondents Who Were Physically and Sexually Abused, Youth Risk Behavior Survey, Florida, 2015 (*n* = 4301).

Population Characteristics	Weighted N ^a^ 542,818	Weighted (%)
Age		
≤14	60,663	11.3
15–17	407,149	74.9
≥18	75,006	13.8
Race		
NH White	267,275	49.2
NH Black	107,290	19.8
Hispanic	168,253	31
Gender		
Male	263,047	48.5
Female	279,771	51.5
Grade		
9th	146,880	27.1
10th	142,889	26.4
11th	129,540	24.1
12th	121,146	22.4
		
Physical Abuse	35,316	6.5
Sexual Abuse	38,351	7.1
		
Both Kinds of Abuse	16,949	3.1
Physical Abuse Only	18,367	3.4
Sexual Abuse Only	21,402	3.9
None	486,100	89.6

NH = non-Hispanic. ^a^ Total is estimated using sampling weights. Unweighted total is *n* = 4301.

**Table 2:** Multiple Logistic Regression Models Predicting Adjusted Odds Ratio (AOR) for Adverse Outcomes among Florida High School Students, by Physical and Sexual Abuse.

	Female		Male	
	AOR	(95% CI)	AOR	(95% CI)
Tobacco				
Both Kinds of Abuse	**8.953**	(5.503, 14.566)	**9.438**	(5.218, 17.071)
Physical Abuse Only	**7.619**	(4.424, 13.121)	**5.447**	(2.843, 10.435)
Sexual Abuse Only	**2.183**	(1.294, 3.683)	1.980	(0.670, 5.853)
None	1.0		1.0	
Alcohol				
Both Kinds of Abuse	**4.164**	(2.574, 6.735)	**4.752**	(2.380, 9.490)
Physical Abuse Only	**4.380**	(2.657, 7.221)	**3.364**	(1.868, 6.058)
Sexual Abuse Only	**2.216**	(1.560, 3.147)	**3.169**	(1.438, 6.987)
None	1.0		1.0	
Marijuana				
Both Kinds of Abuse	**4.520**	(2.970, 6.880)	**5.513**	(3.037, 10.007)
Physical Abuse Only	**5.550**	(3.503, 8.794)	**2.605**	(1.536, 4.419)
Sexual Abuse Only	**2.340**	(1.523, 3.594)	**3.086**	(1.548, 6.154)
None	1.0		1.0	
Suicide Attempt				
Both Kinds of Abuse	**15.199**	(9.533, 24.234)	**34.221**	(16.659, 70.298)
Physical Abuse Only	**4.143**	(2.455, 6.991)	**11.178**	(5.851, 21.356)
Sexual Abuse Only	**4.918**	(3.054, 7.921)	**6.697**	(2.211, 20.283)
None	1.0		1.0	
Depression				
Both Kinds of Abuse	**4.387**	(2.441, 7.883)	**3.834**	(2.169, 6.778)
Physical Abuse Only	**3.479**	(2.005, 6.035)	**2.834**	(1.615, 4.974)
Sexual Abuse Only	**3.551**	(2.458, 5.129)	1.940	(0.815, 4.617)
None	1.0		1.0	

**Table 3:** Multiple Logistic Regression Models Predicting Adjusted Odds Ratio (AOR) for Adverse Outcomes among Florida High School Students, by Physical Abuse and Sexual Abuse.

	Female		Male	
	AOR	(95% CI)	AOR	(95% CI)
Feeling Unsafe				
Both Kinds of Abuse	**5.370**	(2.799, 10.300)	**18.521**	(11.254, 30.479)
Physical Abuse Only	**3.766**	(1.831, 7.745)	**5.883**	(2.978, 11.620)
Sexual Abuse Only	**2.848**	(1.740, 4.663)	**6.927**	(2.906, 16.511)
None	1.0		1.0	
School Performance				
Both Kinds of Abuse	0.842	(0.105, 6.741)	0.253	(0.034, 1.857)
Physical Abuse Only	2.730	(0.745, 10.000)	1.605	(0.726, 3.549)
Sexual Abuse Only	**2.740**	(1.150, 6.529)	+	
None	1.0		1.0	
Inadequate Sleep [<8 h]				
Both Kinds of Abuse	1.182	(0.725, 1.926)	1.408	(0.718, 2.765)
Physical Abuse Only	0.862	(0.483, 1.538)	**2.402**	(1.192, 4.838)
Sexual Abuse Only	**1.882**	(1.214, 2.917)	1.170	(0.553, 2.477)
None	**1.0**		**1.0**	

^+^ Unreliable estimate results of the adjusted ORs are not reported due to failure in convergence.

## Data Availability

Data are contained within the article.
